# One-Step Intraoperative Optical Coherence Tomography Guided Tunnel, Mushroom Femtosecond Laser Big Bubble Deep Anterior Lamellar Keratoplasty

**DOI:** 10.3390/bioengineering11070639

**Published:** 2024-06-22

**Authors:** Mohammed M. Abusayf, Yu-Chi Liu, Evelina Han, Isabelle Lee Xin Yu, Andri K. Riau, Jodhbir S. Mehta

**Affiliations:** 1 Department of Ophthalmology, College of Medicine, King Saud University, Riyadh 11362, Saudi Arabia; 2 Cornea and External Eye Disease Service, Singapore National Eye Center, Singapore 168751, Singapore; liuchiy@gmail.com; 3 Tissue Engineering and Cell Therapy Group, Singapore Eye Research Institute, Singapore 169856, Singapore; evelina.han.j.y@seri.com.sg (E.H.); andri.kartasasmita.riau@seri.com.sg (A.K.R.); 4 Ophthalmology Academic Clinical Program, Duke-NUS Graduate Medical School, Singapore 169857, Singapore

**Keywords:** femtosecond laser, deep anterior lamellar keratoplasty, mushroom configuration, intraoperative optical coherent tomography, intrastromal tunnel

## Abstract

The aim of our study is to investigate the feasibility and outcomes of using a femtosecond laser (FSL) platform (Ziemer LDV Z8) for deep anterior lamellar keratoplasty (DALK), enabling the creation of mushroom-shaped graft–host junctions, lamellar cuts, and intrastromal tunnels, to facilitate the big bubble, in one step. We included wet lab experiments on nine porcine eyes to assess the laser accuracy and cuts depth using an anterior segment (AS) OCT. This was followed by an interventional prospective case series on 10 eyes with variant corneal pathologies. The Z8 system, with in-built intraoperative optical coherence tomography (iOCT), guided corneal scans and directed the cuts. ASOCT showed visible mushroom configurations, lamellar cuts, and tunnels. Deviations from the target were 1.6%, 2.6%, and 3.5%. Anterior lamellar removal was easy in all clinical cases, including corneal scarring. The intrastromal tunnel was found at the preset location and the mushroom configuration was acquired. A big bubble was achieved in all cases. Type 1, 2, and 3 bubbles were formed in eight, one, and one case, respectively. We describe a new approach to DALK in which the in-built iOCT-guided FSL enables safe, precise, controlled, and reproducible desired cuts in one step. The preliminary clinical outcomes were favorable.

## 1. Introduction

Deep anterior lamellar keratoplasty (DALK) is a selective corneal transplantation procedure. It is effective in the treatment of stromal pathologies that spare the Descemet membrane (DM) and corneal endothelium [1]. It can be used for optical, tectonic, or therapeutic indications [2]. DALK is an extraocular procedure with reduced intraoperative risk related to open sky surgery, such as supra-choroidal hemorrhage, iatrogenic intraocular injury, and intraocular infection [3,4]. It provides better postoperative tectonic integrity, less endothelial cell loss, a lower rejection rate, and a longer graft survival rate [1,2,3]. DALK has a shorter healing time, allowing earlier suture removal, hence faster visual recovery and shorter use of topical steroids and fewer steroid-related complications such as cataract and glaucoma [3,4]. Descemetic/Dua’s layer baring DALK using the big bubble (BB) technique has been shown to have similar or better visual and refractive outcomes than mechanically trephined PK [5]. However, BB DALK remains technically challenging and has a steep learning curve. In addition, the success rate of bubble creation varies, depending on the surgeon’s experience and the corneal pathology [2,6].

The introduction of a femtosecond laser (FSL) in penetrating keratoplasty (PK) has made the creation of shaped corneal trephination more standardized, customizable, precise, easier to perform, and less time-consuming than manual techniques [7]. The basic concept of a shaped corneal incision was to hasten visual recovery and improve the mechanical stability of the corneal graft [7]. This is achieved by increasing the surface area between the graft and the host corneal edges, allowing better apposition and faster healing and, hence, earlier suture removal [7]. Additionally, different configurations have been suggested to further enhance the benefits of this incision type according to the underlying pathology [7,8,9]. A top-hat (TH) shaped incision was proposed to supply more donor endothelial cells in corneal endothelial decompensation while maintaining a small anterior graft diameter [8]. In contrast, a mushroom-shaped (MS) incision was introduced to provide a larger refractive surface and peripheral suture placement, which improves the visual outcome. This is achieved while transplanting fewer endothelial cells, thereby reducing the risk of graft rejection [7,8].

The use of an FSL to create a shaped corneal incision in DALK can combine the advantages of a shaped corneal wound, as seen in an FSL-assisted incisional configuration in PK, with the benefits of lamellar surgeries as listed above [10,11,12]. Gadhvi et al. showed that the use of the FSL significantly reduced the risk of intraoperative perforation in comparison to manual DALK [11]. Furthermore, a large diameter DALK (8.5 to 9 mm) has been shown to provide a superior visual outcome and significantly lower astigmatism without increased risk of immune rejection or failure [13,14,15]. It has been shown that MS DALK had similar safety and complications to manually straight-edge trephined DALK, with the advantage of earlier visual recovery due to the large anterior corneal surface [16].

Injecting air, to achieve the BB, at the proper depth using a reproducible technique represents a significant surgical challenge, especially for novice corneal surgeons [17]. Recently, FSL platforms have allowed the creation of a lamellar cut and an intrastromal tunnel to help achieve the BB [18]. The success rate of achieving the BB using different manual surgical techniques averages around 36–85% [2,6,18]. Lucisano et al. reported a 100% success rate of achieving a BB straight cut DALK with an intrastromal tunnel created using the Victus FSL platform (Bausch & Lomb, Bridgewater, NJ, USA) [19]. Likewise, Liu et al. showed a similar outcome of a 100% success rate in achieving the BB using a Ziemer LDV Z8 FSL platform (Ziemer Ophthalmic System, Port, Switzerland). In addition, it has been shown that the use of an FSL-created intrastromal tunnel has shown a lower rate of conversion to PK [20].

The next stage of the evolution of the straight cut, intrastromal tunnel is to combine this with a mushroom cut. However, this may be challenging due to the creation of a deep tunnel cut beneath two lamellar cuts. In this case series, we report the results of the simultaneous creation of an MS DALK with an intrastromal tunnel creation in one step using the low-energy Ziemer LDV Z8 FSL platform (Ziemer Ophthalmic System, Port, Switzerland) with guidance of its in-built, real-time intraoperative OCT.

## 2. Materials and Methods

Our study was divided into a preclinical part and a clinical part to ensure its feasibility and accuracy first and then its clinical effectiveness.

### 2.1. Porcine Eye Study

Nine fresh porcine eyes were used to assess the accuracy in creating the deep anterior vertical and lamellar cuts, to form a mushroom’s cap and stalk configuration, as well as the intrastromal tunnel in the residual stromal bed to aid in BB formation. After application of the laser head and applanating the corneal surface, the in-built, real-time OCT imaged the cornea, and the laser energy settings and cutting parameters were programmed.

**Figure 1:**
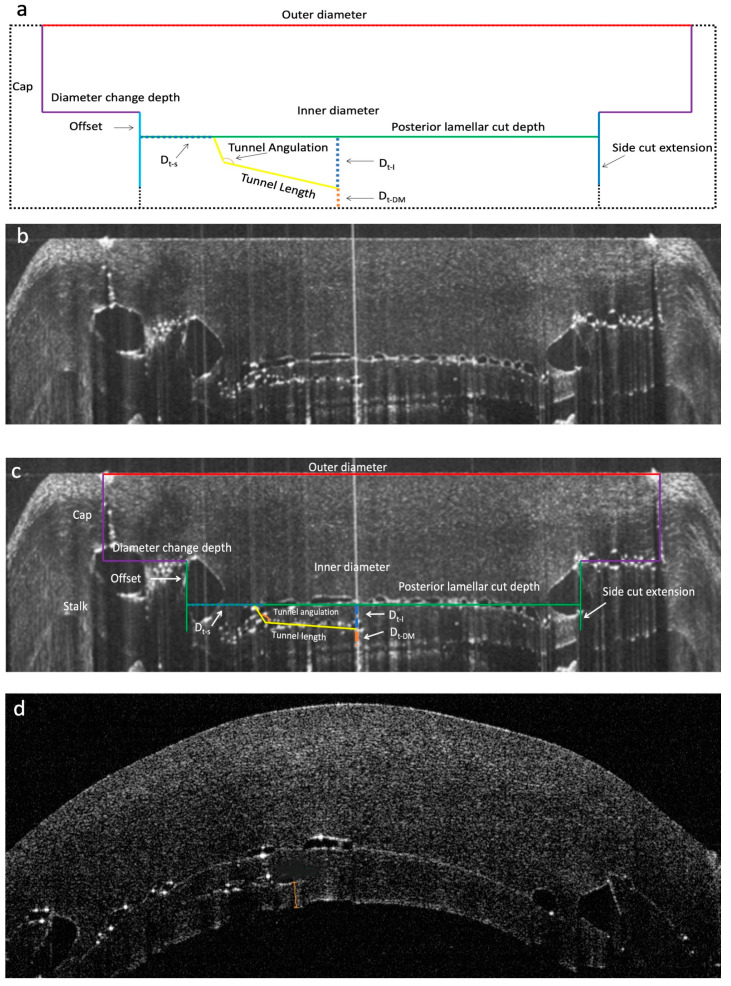
Mushroom configuration cuts with intrastromal tunnel of the host cornea of fresh porcine eye. (**a**) A diagram of the applanated cornea illustrating parameters. (**b**,**c**) iOCT photo from Z8 laser machine without “b” and with “c” illustrating parameters. (**d**) Immediately post-cut OCT obtained using Optovue (non-applanated surface) showing the corresponding cut.

The laser cutting speed and energy were 8.0 mm/s and 120% for the lamellar cut, 5.00 mm/s and 105% for the tunnel cut, and 40 mm/s and 130% for the side cuts, respectively. The laser cutting parameters were programmed as follows. The outer and inner diameters were set to 8.8 mm and 6 mm, respectively. The diameter change depth, i.e., the “cap”, corresponding to the anterior part of the vertical side cut and ring lamellar cut (anterior lamellar cut), was set to 500 µm. The “stalk” thickness was the remaining stroma from the end of the diameter change depth till the DM at the thinnest point. It was divided by the posterior lamellar cut into two components, an offset and a residual stromal bed. The reference point for all measurements was the DM at the thinnest point. The posterior lamellar cut was adjusted based on iOCT measurements, aiming to leave a residual stromal bed of at least 200 µm from the DM at the thinnest point (Dl-DM). The distance between the posterior lamellar cut and the tunnel end (Dt-l) was set to 100 µm, the tunnel length was 2 mm with an angle of 60 degrees, and the distance from the posterior side cut of the lamellar to the tunnel (Dt-S) was 1 mm; the tunnel width was 0.4 mm, and the distance between the tunnel end and DM (Dt-DM) was 100 µm. The tunnel was positioned at 90 degrees meridian at 12 o’clock. The posterior part of the side cut was extended beyond the posterior lamellar cut but was less than the DM at the thinnest point. The expected results were displayed in real-time iOCT before starting the cut. A diagram illustrating the laser cutting parameters is shown in Figure 1a–c. All the above parameters were adjustable.

#### Evaluation of Cutting Accuracy with ASOCT

After completion of the procedure, re-imaging of the non-applanated cut corneas was performed using an anterior segment OCT (ASOCT; RTVue, Optovue). The intended parameters on iOCT and achieved programmed laser parameters on the postoperative OCT including diameter change depth, posterior lamellar cut depth, distance from the posterior side cut of the lamella (Dt-S), and distance between the tunnel end and DM (Dt-DM) were compared to assess the accuracy (Figure 1d).

### 2.2. Case Series

In this study, 9 patients with a total of 10 eyes were included. Three patients had keratoconus (KC), while 2 patients had lattice corneal dystrophy (LCD), 1 had type 1 granular corneal dystrophy (GCD1), and 2 had type 2 granular corneal dystrophy (GCD2) [21]. Additionally, one patient had systemic mucopolysaccharidosis (MP) with Morquio-Brailsford syndrome. The last patient had the procedure in both eyes sequentially. One of the KC eyes had significant apical scarring. All patients had ASOCT (RTVue, Optovue Inc., Fremont, CA, USA) and Pentacam (Oculus, Wetzlar, Germany) performed preoperatively. The preoperative best corrected visual acuity (BCVA), mean keratometry (Km), central corneal thickness (CCT), thickness at the thinnest point within the 6 mm area, and white to white measurements were documented.

The patients underwent FSL-assisted mushroom configuration DALK with an intrastromal tunnel, using the LDV Z8 platform by a single surgeon (JSM) under general (GA) or local anesthesia with sedation. 

The center of the cornea was marked with a marking pen. A two-step docking procedure was performed, to ensure accurate centration. The suction ring was first applied and after a vacuum was obtained, the flat applanating handpiece of the Ziemer LDV was mounted on the ring, and the patient’s cornea was carefully applanated. Once the cornea was applanated and aligned, the in-built OCT performed a horizontal and a vertical scan for surgical planning. The thinnest point within the 6 mm area was measured intraoperatively using iOCT. The applanation time and total cut time were recorded.

The laser cutting speed and energy were 8.0 mm/s and 130% to 140% for the lamellar cut, 5.00 mm/s and 105% to 110% for the tunnel cut, and 40 mm/s and 140% for the side cuts, respectively. The indicated upper limit of the laser energy was used in cases with dense corneal opacities. The suction ring size used was 10 mm. The vacuum was 700 mbar. The host programmed dimensions are shown in Table 1. The reference point for all measurements was the DM at the thinnest point. The posterior lamellar cut was adjusted intraoperatively, based on iOCT measurements, aiming to leave a residual stromal bed of at least 150 µm from the DM at the thinnest point. The distance between the residual stromal bed and tunnel end (Dt-l) was 100 µm, the tunnel length was 2 mm with an angle of 30 to 60 degrees, and the distance from the posterior side cut of the lamella to the tunnel (Dt-s) was 1 mm; the tunnel width was 0.4 mm and the distance between the tunnel end and the DM (Dt-DM) was 50 µm. The tunnel was positioned at 90 degrees meridian at 12 o’clock. The posterior part of the side cut was extended beyond the posterior lamellar cut but was less than the DM at the thinnest point.

**Figure 2:**
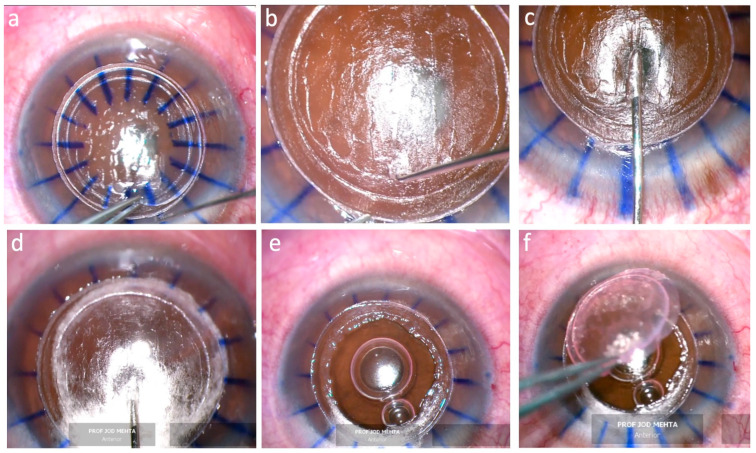
Intraoperative photos of recipient preparation. (**a**) The mushroom cap and the offset part of the stem are removed as a single piece. (**b**) A Sinskey hook is used to easily find and open the tunnel in the residual stromal bed. (**c**) A canula is introduced into the tunnel. Notice the almost flat position of the cannula to avoid ripping the tunnel with the cannula shaft. (**d**) A type 1 BB was formed after air injection into the tunnel. (**e**) A bare DM following stromal removal. (**f**) The mushroom-shaped donor is placed.

Following the laser resection, the anterior lamella was removed using a forceps and a Sinskey hook to aid in bridge dissection if present (see Figure 2a). The tunnel was identified and opened using a Sinskey hook (Figure 2b). Then, injection of air through the intrastromal tunnel was performed by inserting and advancing a Tan 27G DALK cannula (ASICO, Parsippany, NJ, USA, AE-7803) connected to an air-filled 5-mL syringe (Figure 2c,d). Once a bubble was achieved, the procedure was completed in a similar fashion to the conventional big-bubble procedure [1]. A sharp 30-degree blade was used to puncture the stromal wall of the bubble and Anwar corneal scissors (Duckworth and Kent, Baldock, UK, 1-218 and 1-219) were used to cut the residual stroma into quadrants and excise it within the laser trephination cut (Figure 2e).

**Figure 3:**
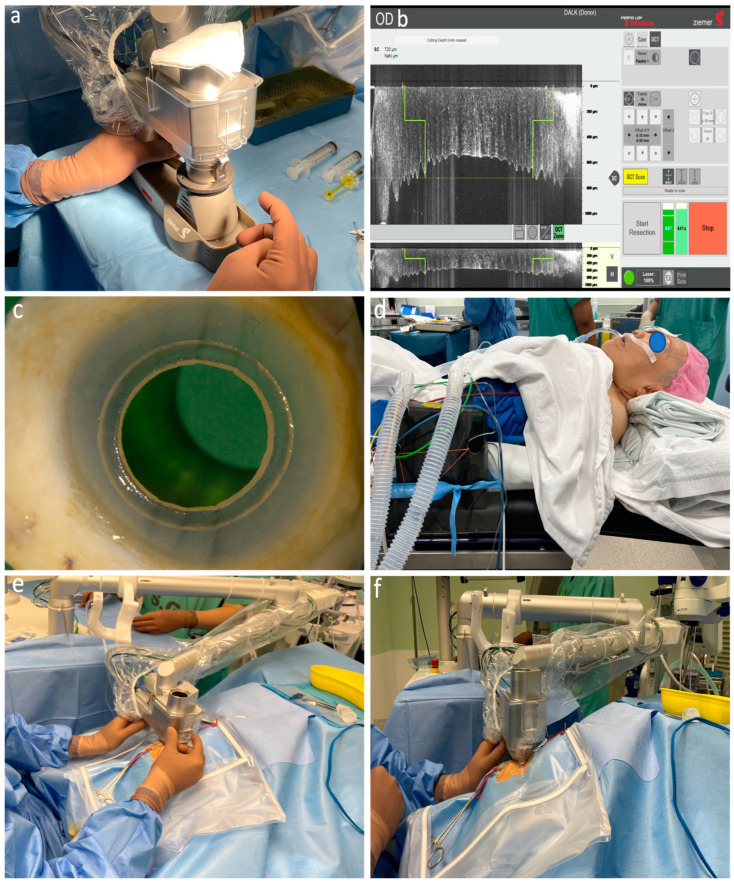
Donor and recipient docking and laser cutting procedures. (**a**) The donor cornea is mounted onto the artificial anterior chamber. The laser handpiece is then docked to image and deliver programmed cutting. (**b**) iOCT photo from Z8 laser platform showing donor parameters adjustment. Full-thickness donor cutting occurs after determining the host matching stalk length. (**c**) Remaining corneoscleral rim showing full-thickness mushroom configuration cut. (**d**) Side view photo of the patient with MP showing short stature and difficult posturing. The patient’s head does not reach the edge of the surgical table. (**e**,**f**) Docking of the same patient. Notice the distance between the surgeon and the patient’s head and eye.

The donor tissue was then trephined using the LDV Z8 platform with the Ziemer artificial anterior chamber (Figure 3a). The central cornea thickness (CCT) was measured intraoperatively using iOCT (Figure 3b,c). The donor anterior outer (donor size) and posterior inner diameters were programmed to be 0.1–0.2 mm and 0.1 mm larger than the host, respectively. Scraping the thickened epithelium of the old donor cornea allowed easier laser penetration and dissection. Donor folds can be encountered during applanation due to low irrigation flow in the artificial chamber system. Raising the irrigation flow by increasing the bottle height, applying pressure using a pressure cuff or an artificial chamber pressurizer, or injecting viscoelastic material under the donor help to overcome donor folds. After removal of the donor DM, the corneal button was then placed onto the recipient bed (Figure 2f). Finally, the graft was secured with 16 interrupted 10-0 nylon sutures. A short video of the procedure, including the laser cutting, is shown in the Supplementary Material (Video S1).

The postoperative best corrected visual acuity (BCVA), mean keratometry (Km), central corneal thickness (CCT), follow-up duration, and time of elective suture removal were documented at the last follow-up.

The STROBE cohort reporting guidelines were used [22].

### 2.3. Statistical Analysis

All values were expressed as median (range, min–max) unless specified. The difference between the intended and achieved laser cutting parameters was calculated using a percentage of deviation for each parameter. The visual acuity was converted to the mean logarithm of the minimum angle of resolution (LogMAR) value for statistical analysis. A two-tailed Wilcoxon signed-rank test was used to compare preoperative and postoperative visual acuity (VA). A *p*-value of less than 0.05 was considered statistically significant. All statistical analyses were performed using Microsoft Excel. 

## 3. Results

### 3.1. Porcine Eyes

On ASOCT scans, the diameter change depth, lamellar cut and tunnel track were visible (Figure 1d). The mean diameter change depth, Dl-dm, and Dt-dm were 492.23 ± 8.5 µm, 205.1 ± 15.15 µm, and 103.5 ± 11.5 µm measured using ASOCT, respectively, indicating that the deviations from the intended target were 1.6%, 2.6%, and 3.5%, respectively. 

### 3.2. Case Series

**Figure 4:**
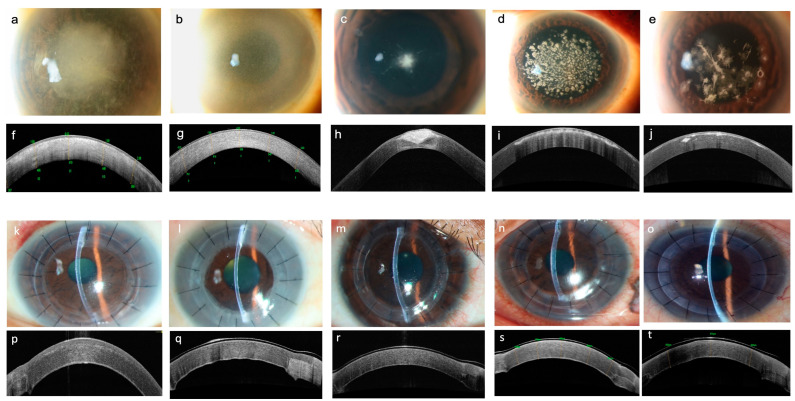
Corneal opacities encountered from several corneal pathologies. Preoperative photos and OCT: (**a**,**f**) LCD. (**b**,**g**) MP. (**c**,**h**) KC with apical scarring. (**d**,**i**) GCD1. (**e**,**j**) GCD2. Early postoperative photos and OCT of the same cases: (**k**,**p**) LCD. (**l**,**q**) MP. (**m**,**r**) KC with apical scarring. (**n**,**s**) GCD1. (**o**,**t**) GCD2.

Ten eyes of nine patients were included in this study with a median age of 53 (19–71). There were five females and four males. The median best corrected visual acuity (BCVA) in LogMAR was 0.70 (0.40–1.00). The median of mean keratometry (Km) for all diagnoses was 43.7 diopters (range, 41.5–69.0 diopters), while the median Km for the KC group was 62.0 diopters (range, 53.0–69.0 diopters). The median central corneal thickness (CCT) and median thinnest point within the 6 mm area was 535 µm (356–628 µm) and 535 µm (325–617 µm), respectively. The median thinnest point within the 6 mm area measured intraoperatively was 557 µm (322–608 µm). The median white to white (WTW) measurement was 11.80 (11.50–13.00). The median anterior trephination diameter of the host cornea was 8.20 mm (8.00–8.40 mm). The median CCT for donor corneas measured intraoperatively was 513 µm (461–629 µm). The median applanation time and total cut time were 1.0 min (46–69 s) and 4.7 min (3.3–8.6 min), respectively. In all cases, the mushroom configuration was achieved in both recipient and donor tissues and all cuts were complete. The anterior stromal lamella was removed with minimal difficulty, even in patients with corneal scars (Figure 4). The intrastromal tunnel could be found at the preset location of 12 o’clock. The big bubble was achieved in all cases. A type 1 bubble was formed in eight cases. Type 2 and 3 bubbles were formed in two KC cases. There were no intraoperative complications. 

Postoperatively, case 1 had a double anterior chamber early postoperatively. However, no intervention was necessary and the detachment resolved spontaneously. The median follow-up duration was 4.5 months (1 week–10 months). On the last follow-up, the median postoperative BCVA in LogMAR showed significant improvement to 0.30 (0.00–0.70) (*p* = 0.005). Except for one case, all patients in this series had cataracts in different densities. Several patients were planned for cataract surgery once the refractive outcome was stable. The postoperative median Km for all diagnoses measured was reduced to 46.5 diopters (range, 45.5–47.0 diopters). The postop median CCT was 513 µm (461–624 µm), and the postop median Km for the KC group was 46.5 diopters (range, 46.4–47.0 diopters). In addition, an improvement in corneal irregularity indices was noticed on the last follow-up. Elective removal of the sutures was started at a median of 3 months (2–5 months) postoperatively.

## 4. Discussion

FSL-assisted DALK refers to the use of an FSL to trephine and shape the graft–host junction, creating a lamellar plane, to then try to achieve a BB via a manual created intrastromal tunnel [7]. The purpose of using a laser was to reduce intraoperative complications (of which perforation and conversion to PK are the least desirable), standardize and shorten the procedure, reduce the learning curve, and improve the refractive outcome [7,23]. Different FSL platforms and various techniques have been studied over recent years [7]. The recent advancement in this field has been the introduction of an FSL-assisted, OCT-guided intraoperative tunnel creation [6,24]. This offers a significant leap in order to standardize this difficult surgery. We have previously shown the efficacy of a straight-edge trephination with the creation of an intrastromal tunnel using the Z8 platform [6]. In the present study, we describe its use in a mushroom configuration, offering the refractive advantages of the mushroom cut, with the simultaneous advantages of a laser tunnel creation.

The iOCT on the Z8 platform allows the surgeon to program and visualize the corneal cuts in real time. It aids in accurately adjusting the vertical and lamellar cuts of the mushroom configuration and precisely determines the location of the tunnel end, safely from the DM [6]. In addition, the complex combination of mushroom cuts, where two vertical and two lamellar cuts as well as the intrastromal tunnel was achieved in one step using one license per recipient via dedicated software without repeated docking. Buzzonetti et al. have reported the use of 60 kHz IntraLase FSL (Advanced Medical Optics, Inc., Santa Ana, CA, USA) to create an intrastromal channel 50 µm above the DM and a shaped trephination in a series of adults/children with keratoconus or congenital opacities [25]. The described technique staged the laser cutting where three and two procedures (licenses) for the recipient and donor preparation were used, consecutively, increasing costs and operating time. The Victus FSL (Bausch & Lomb, Bridgewater, NJ, USA) has also been used to create intrastromal tunnels in one step [24]. In a series on cadaveric corneas, the authors showed the benefits of iOCT and real-time adjustment of the resection planes [24]. The authors used dedicated software that allowed for the creation of sequential corneal incisions, including shaped trephination in one step without the need for repeated docking. As yet, no clinical cases have been reported.

The Z8 creates the tunnel cut first followed by the remaining corneal cuts. This has been shown to increase the success of achieving a big bubble [24]. In our porcine study, the agreement between the targeted and achieved cuts showed good accuracy of laser cutting. The deviations from the intended target for the cap, posterior lamellar cut and tunnel end were small, at 1.6%, 2.6%, and 3.5%, respectively. In addition, the trend of iOCT measurements of the thinnest point was lower than preoperative measurements. Accuracy is especially important in KC cases using a flat applanating system, where the thinnest point was accurately determined to prevent accidental perforation during laser tunnel creation. The risk of FSL perforation might be even higher in FSL platforms that depend on corneal thickness measurements from preoperative investigations, i.e., without real-time iOCT. A case of micro-perforation has been reported during tunnel creation by an IntraLase due to the use of preoperative corneal thickness scans [20]. There were no reported cases of corneal perforation in this series or any of the published series with the Z8 platform [6,18].

Both above-mentioned platforms (IntraLase and Victus) are within the microjoules range. Creating a tunnel at this depth has been shown to have an effect on the endothelium [26]. The LDV Z8 laser uses a low-energy concept (nanojoule range) and is, hence, safer when the tunnel is closer to the endothelium, i.e., at 50 µm in our cases [27]. Furthermore, the mobility and footprint size of the Z8 is an advantage since it avoids the need to transfer the patient from the laser suite to the OT and allows all the procedures to be performed under GA, which is useful in children, uncooperative patients, and patients with specific musculoskeletal abnormalities. One of our patients in this series had systemic MP and had posture and mobility limitations (Figure 3d–f). This reduced the overall risk and shortened the time of the surgery. The postoperative clinical results on the last follow-up in this series were similar to the literature where a customized graft–host junction was used [25]. The BCVA improved significantly at 4.5 months (1 week–10 months).

In all clinical cases, the anterior stromal lamella was removed without difficulty even in patients with corneal scars. The mushroom configuration was achieved and the intrastromal tunnel could be found and opened at the preset location using a Sinskey hook followed by cannula insertion. Care was taken not to significantly tilt the cannula tip downward to avoid ripping the tunnel with the shaft leading to air leakage (Figure 2c,d). The big bubble was achieved in all cases. However, a type 2 and type 3 bubble were formed in two cases, respectively. The surgeon elected to continue with manual dissection in the type 2 bubble case to reduce the risk of perforation [2]. This was facilitated using the laser-created posterior lamella plane as a starting point and reference plane to further manually dissect down to the pre-DM depth. In KC cases, it was important that the posterior part of the donor cornea (mushroom stalk) was matched with the recipient bed. The host cornea was cut first to ensure that the diameter change depth (mushroom cap) was programmed to leave a stalk length of at least 200 µm, of which, 50 µm (offset) of the stalk was removed after the posterior lamellar cut, leaving a minimum of 150 µm residual stroma for tunnel creation (Figure 1a–c). This was followed by cutting the donor by simply adjusting the diameter change depth in relation to the donor thickness so the desired length of the stalk can be achieved (Figure 3b; Table 2 (Case 6, example of matching stalk thickness between donor and recipient)). This is important, especially when using donor tissue that is from a longer duration of storage, and in those with low endothelial cell counts which are more swollen, as in the cases in this series. Matching the cap thickness between the donor and the recipient is not crucial because the donor cap will be compressed by the sutures and allows restoration of corneal stromal thickness.

The wavy contour of the DM, as shown on the intraoperative OCT scans (Figure 3b), resulted from the applanation effect of the Ziemer flat interface, and this may be more obvious in patients with keratoconic corneas because of a deformation from the applanation on thinner, steeper, and less rigid cornea [6]; hence, careful calculations must be made in using this technique in steep corneas, which may induce distortion from the applanation. The highest preop Km in the KC group in our series was 69.0 diopters. A further advancement in FSL-assisted DALK would be the use of a liquid patient interface (LPI) where no applanation is applied. A LPI allows the natural curvature of the cornea to maintain its shape, avoiding mechanical compression, which will be especially useful in ectatic corneas, improving the side cuts geometrics at the graft–host junction, and reducing the IOP rise [28]. The Z8 has a liquid hand piece and software is currently available for liquid PK, which could be adapted for liquid DALK [28,29]. A clear improvement in corneal topographic irregularities was noticed, especially in KC cases. This study has limitations due to the short follow-up period, which prevents reporting on refractive outcomes and endothelial cell counts. These data will be included in a future publication with a longer follow-up period. Additionally, the sample size is 10 eyes. However, the primary goal of this study is to describe a new technique using a mushroom configuration with an intraoperative guided tunnel. Therefore, this pilot study aimed to demonstrate the efficacy and feasibility of performing this technique. As discussed above, previous feasibility studies have had small sample sizes or were conducted only on experimental models such as cadaveric or non-human corneas. The strengths of this study include a two-stage design. The initial in vitro investigation on porcine eyes establishes the precision of the laser cuts, thereby mitigating potential risks before human application. Subsequently, a clinical case series assesses the feasibility and safety of the technique in a diverse range of corneal pathologies. This sequential approach, coupled with the triangulation of preclinical and clinical data, strengthens the research design and lays a foundation for further investigation of this DALK method. The aim of this study was to show the feasibility, safety, and efficacy of this technique—mushroom tunnel DALK. This study is limited by the short follow-up period, which precludes reporting on refractive outcomes and endothelial cell counts. These data will be presented in a future publication with a longer follow-up period. Additionally, the sample size is 10 eyes. However, the primary goal of this study is to describe a new technique using a mushroom configuration with an intraoperative guided tunnel. Therefore, this pilot study aimed to demonstrate the efficacy and feasibility of performing this technique. As discussed above, previous feasibility studies have had small sample sizes or were conducted only on experimental models such as cadaveric or non-human corneas. 

## 5. Conclusions

In conclusion, we describe a new approach to DALK in which the in-built iOCT guided low-energy FSL was able to provide safe, precise, controlled, and reproducible mushroom lamellar and tunnel cuts to a desired depth in one step, facilitating the injection of air for the big-bubble DALK procedure. The iOCT imaging allowed real-time adjustments of parameters, aiding in surgical planning. The preliminary clinical outcomes were favorable. The success rate of achieving an MS tunnel DALK and big bubble in our series was 100%.

## Figures and Tables

**Table 1:** Host programmed laser parameters.

Host	Thinnest Point iOCT (µm)	Diameter Change Depth (cap) (µm)	Lamellar Cut Depth (µm)	Residual Bed Thickness (µm)	Stalk Thickness (µm)	WTW (mm)	Anterior Diameter (Donor Size) (mm)	Posterior Diameter (mm)	Total cut Time (seconds)	Applanation Time (seconds)
Case 1: LCD	396	190	245	151	190	11.80	8.00	6.50	48	308
Case 2: KC	387	225	235	152	162	13.00	8.00	6.50	48	518
Case 3: MP	565	245	411	154	320	11.80	8.30	6.00	60	275
Case 4: KC	322	103	153	169	219	12.00	8.30	6.50	46	196
Case 5: KC	425	150	270	155	275	12.00	8.20	6.50	53	327
Case 6: LCD	589	380	396	193	209	12.10	8.00	6.50	61	292
Case 7: GCD 2	608	350	455	153	258	11.70	8.20	6.00	65	232
Case 8: GCD 2	557	350	405	152	207	11.50	8.20	6.00	61	239
Case 9: GCD 1	573	350	420	153	223	11.70	8.00	6.00	69	252
Case 10: MP	565	300	411	154	265	11.80	8.40	6.00	63	290

iOCT: intraoperative optical coherence tomography. WTW: White to white. LCD: Lattice corneal dystrophy. KC: Keratoconus. MP: Mucopolysaccharidosis. GCD: Granular corneal dystrophy.

**Table 2:** Donor programed parameters of Case 6.

Donor	iOCT Thickness (µm)	Predetermined Stalk Thickness (µm)	Adjusted Diameter Change Depth (cap) (µm)	Anterior Diameter (mm)	Posterior Diameter (mm)
Case 6	507	209	298	8.10	6.60

iOCT: intraoperative optical coherence tomography.

## Data Availability

The raw data supporting the conclusions of this article will be made available by the authors on request.

## Supplementary Materials

The following supporting information can be downloaded at: https://www.mdpi.com/article/10.3390/bioengineering11070639/s1, Video S1: A short video of the procedure, including laser cutting.
